# Supplementary material for: Aspirin: A Suicide Inhibitor of Carbonic Anhydrase II
Source: Biomolecules. 2020 Mar 31;10(4):527. doi: 10.3390/biom10040527 (PMC7226357; doi:10.3390/biom10040527)
Supplement: Supplementary file 1 [file biomolecules-10-00527-s001.pdf]

Supplementary

Table S1. X-Ray Statistics.

Statistic	CA II Salicylic Acid
**Wavelength (Å)**	0.9795
**Resolution range (Å)**	35.55 - 1.36 (1.40 - 1.36)
**Space group**	P 1 21 1
**Unit cell: a,b,c (Å)**<br>**α, β, γ (°)**	41.3, 42.4, 72.0<br>90, 104.2, 90
**Total reflections**	145498 (7504)
**Unique reflections**	48888 (3558)
**Multiplicity**	3.0 (2.1)
**Completeness (%)**	93.72 (66.69)
I/I_σ	11.6 (0.8)
**Wilson B-factor (Å^2)**	15.85
R_{merge}[a] **(%)**	4.54 (77.60)
R_{work}[b] **(%)**	15.44 (32.61)
R_{free}[c] **(%)**	16.67 (33.08)
R_{pim}[d] **(%)**	2.97 (58.68)
**Reflections used in refinement**	49449 (3514)
**Reflections used for R-free**	2002 (138)
**Number of non-hydrogen atoms**	2359
**macromolecules**	2128
**ligands**	55
**solvent**	176
**Protein residues**	258
**RMS(bonds) (Å)**	0.012
**RMS(angles) (°)**	1.21
**Ramachandran favored (%)**	97.7
**Ramachandran allowed (%)**	2.3
**Ramachandran outliers (%)**	0.0
**Rotamer outliers (%)**	1.3
**Average B-factor(Å^2)**	22.4
**macromolecules**	21.5
**ligands**	34.6
**solvent**	30.7

[a]$R_{merge} = (\Sigma|I - \langle I\rangle|/\Sigma \langle I\rangle) \times 100$.

[b]$R_{work} = (\Sigma|F_o - F_c|/\Sigma|F_o|) \times 100$.

[c]R_{free} is calculated in the same way as R_{cryst} except it is for data omitted from refinement (5% of reflections for all data sets).

[d]$R_{pim} = [(\Sigma\sqrt{1/N} - 1)\Sigma|I - \langle I\rangle|/\Sigma \langle I\rangle] \times 100$.

Values in parentheses correspond to those of the highest-resolution shell.
